# Acceptability of Web-Based Mental Health Interventions in the Workplace: Systematic Review

**DOI:** 10.2196/34655

**Published:** 2022-05-11

**Authors:** Johanna Scheutzow, Chris Attoe, Joshua Harwood

**Affiliations:** 1 Department of Psychosis Institute of Psychiatry, Psychology and Neuroscience, School of Academic Psychiatry King’s College London London United Kingdom; 2 Maudsley Learning, South London & Maudsley NHS Foundation Trust London United Kingdom

**Keywords:** acceptability, e-mental health, online mental health interventions, occupational online interventions, employees, mobile phone

## Abstract

**Background:**

Web-based interventions have proven to be effective not only in clinical populations but also in the occupational setting. Recent studies conducted in the work environment have focused on the effectiveness of these interventions. However, the role of employees’ acceptability of web-based interventions and programs has not yet enjoyed a similar level of attention.

**Objective:**

The objective of this systematic review was to conduct the first comprehensive study on employees’ level of acceptability of web-based mental health interventions based on direct and indirect measures, outline the utility of different types of web-based interventions for work-related mental health issues, and build a research base in the field.

**Methods:**

The search was conducted between October 2018 and July 2019 and allowed for any study design. The studies used either qualitative or quantitative data sources. The web-based interventions were generally aimed at supporting employees with their mental health issues. The study characteristics were outlined in a table as well as graded based on their quality using a traffic light schema. The level of acceptability was individually rated using commonly applied methods, including percentile quartiles ranging from low to very high.

**Results:**

A total of 1303 studies were identified through multiple database searches and additional resources, from which 28 (2%) were rated as eligible for the synthesis. The results of employees’ acceptability levels were mixed, and the studies were very heterogeneous in design, intervention characteristics, and population. Approximately 79% (22/28) of the studies outlined acceptability measures from high to very high, and 54% (15/28) of the studies reported acceptability levels from low to moderate (overlap when studies reported both quantitative and qualitative results). Qualitative studies also provided insights into barriers and preferences, including simple and tailored application tools as well as the preference for nonstigmatized language. However, there were multiple flaws in the methodology of the studies, such as the blinding of participants and personnel.

**Conclusions:**

The results outline the need for further research with more homogeneous acceptability studies to draw a final conclusion. However, the underlying results show that there is a tendency toward general acceptability of web-based interventions in the workplace, with findings of general applicability to the use of web-based mental health interventions.

## Introduction

### Background

There is an increasing level of awareness regarding the importance of health and well-being in the workplace [1]. Anxiety, stress, and depression are the dominant mental health issues for workers in the United Kingdom, with a prevalence of 1320 cases per 100,000 workers, causing close to 18 million lost working days per year [2]. Employers have a responsibility to take care of their employees and provide support for both their physical and mental health [3,4].

Web-based mental health interventions are increasingly being used in the work environment as they have the advantage of being cost-effective, efficient, anonymous, location-independent, flexible, and empowering. They are regularly used for both prevention and intervention [5-10].

Web-based interventions also have multiple flaws, including technical difficulties, ethical concerns, increased attrition rates, and low engagement in the absence of guidance by professional support [6,11]. Therefore, it is important to understand the barriers that reduce engagement and acceptability of web-based interventions. Multiple systematic studies provide evidence of the effectiveness of web-based mental health interventions at work [12,13]. Importantly, they outline the need to tailor interventions to populations’ needs, which requires greater insight into barriers and the acceptability of web-based mental health interventions in the workplace.

The acceptability of an intervention includes users’ emotional and cognitive responses to the intervention [14], including affective perceptions, burden and barriers, perceived benefits, understanding of the intervention, opportunity costs, and usability. In practice, this takes into account the individuals’ preferences for features and tools, their willingness to use web-based interventions, their engagement (eg, dropout and attrition rate), and users’ perceived utility or satisfaction with the intervention.

Studying users’ acceptability of new treatments has ethical, methodological (validity), and practical applications [15]. Specifically, ethical obligations include the exploration of reasons for acceptable or unacceptable treatments as perceived by the users. It is important to understand potential barriers to intervention engagement before introducing the intervention to employees. Awareness of intervention efficacy alone does not mean that employees accept web-based interventions as a useful tool for self-help.

Sekhon et al [14] outlined studies assessing interventions’ acceptability by using operational definitions in line with measurable acceptability data (dropout rate and satisfaction rating) and qualitative studies focusing on in-depth user experiences. Current research has been limited to studies on clinical populations. Clinical populations differ significantly in symptom severity, level of risks, functionality, and response to treatment; thus, the results might not be generalizable to occupational populations [13]. Therefore, it is relevant to explicitly assess employees’ acceptability of web-based interventions.

### Objectives

This systematic review aimed to assess employees’ acceptability of web-based interventions to improve their mental health. The study aimed to inform intervention design and utility by evaluating user experience and barriers and facilitators to using web-based mental health interventions in the workplace.

## Methods

This systematic review was conducted in line with the PRISMA (Preferred Reporting Items for Systematic Reviews and Meta-Analyses) guidelines [16] and followed the ENTREQ (Enhancing the Transparency in Reporting the Synthesis of Qualitative Research) guidelines [17,18].

### Eligibility Criteria

Eligible studies met the Population, Intervention, Comparison, Outcome criteria and included qualitative interviews, quantitative studies including scale measures of satisfaction and forms of attrition rates, and mixed methods studies. Acceptability was assessed by means of both direct (acceptability, satisfaction, and experience) and indirect (compliance, completion, adherence, attrition, and dropout rate) measures. Studies were included if they were available in English and published after 2005.

#### Population

The population was narrowed down to people aged ≥18 years as there is a difference between child and adult interventions. The participants could be employed part-time or full-time or self-employed. Studies were included if the participants were >60% employed. This threshold guaranteed that the main outcome could be generalized to the eligible population for the study’s purpose.

#### Intervention

Following the guidance of the meta-review by Joyce et al [19] on general workplace mental health interventions, web-based interventions were kept broad to include those that were conducted at work, had a work-related component, or aimed to treat work-related risk factors (eg, stress, depression, or anxiety). However, eligible interventions had to be exclusively web-based programs or interventions that targeted employed people or were applied in an occupational setting. Interventions or programs could be delivered via a computer program, app, or website. They could also differ in the device used to deliver the content (computer, laptop, or mobile phone) as well as include various forms of multimedia. All interventions aimed to change employees’ behavior or mental health. They could have the aim of preventing, treating, or rehabilitating mental health issues.

#### Comparison

This review compared randomized controlled trials (RCTs), nonrandomized comparative trials, noncomparative trials, explorative studies, and qualitative studies published between 2005 and 2019.

#### Outcome

Studies were included if they measured acceptability directly or indirectly by means of qualitative assessment of acceptability, satisfaction, and experience or the indirect measure of acceptability through compliance, completion, adherence, attrition, or dropout rate. Studies were included that assessed the potential willingness to use interventions or the potential features of interventions that were preferred or addressed as disadvantageous for utility.

### Exclusion Criteria

Studies were excluded if they did not meet the Population, Intervention, Comparison, Outcome criteria; that is, if they included guidance through coaches, therapists, or face-to-face interactions and were applied to participants who were retired or unemployed (>40%). In addition, studies were excluded if they did not measure *acceptability* or *willingness of use* as an outcome variable or used interventions that were not focused on the users’ mental health.

### Data Sources

The search was conducted in July 2019 and included the following electronic databases: PsycINFO (Ovid), Embase (Ovid), MEDLINE (Ovid), Global Health (Ovid), and the Cochrane Library Trials (CENTRAL). Backward searching was used to ensure that no key papers were missed.

### Search Strategy

Databases were searched for studies published between 2005 and 2019. Duplicates were removed (Ovid search option). The Boolean system was applied using AND and OR (Textbox 1) to combine different terminologies of 4 key concepts included in a free-text and keyword search. Specific occupations were added to the general search of employees to increase the likelihood of finding studies on high stress–exposed work settings; for example, in the military or firefighter professions. The search terms used were categorized as occupational settings (employee), web-based interventions, mental ill health, and acceptability of interventions. All the key terms considered American and British spelling.

**Search terms organized into 4 concepts.**

**Concepts (combined using *AND*) and corresponding search terms (combined using *OR*)**

- Employee: employment, job, work, worker*, workplace, occupation*, employee*, manager*, line-manager*, staff, military, fire-fighter*, police, emergenc*, business, organisational*, work related, personnel
- Web-based intervention: video-therap*, mobile therap*, computerised cbt, ccbt, digital therap*, e-mental health, e-health, ehealth, computerised* therap*, internet-based intervention, occupational e-therap*, occupational e-mental health, e-therap*, web-based therap*, internet based therap*, online* therap*, tele-medicine, tele-therap*, tele-psychiatry, tele-psycholog*, computer-assisted therap*, electronic intervention, smartphone intervention online, psychological*, app
- Mental ill health: stress, mental health, mental illness, mental disorder, depress*, anxiety, affective symptoms, burnout, resilience wellbeing, workplace wellbeing, ptsd, trauma, acute stress disorder, return to work, psychological*, sick* leave*, sick* day*, sick* absen*, absenteeism, emotional stress, interpersonal stress, life stress, mental stress, chronic stress, job stress
- Acceptance: accept*, willing*, open*, attitude, feasab*, satisfaction compliance, reasons for drop out, drop-out, utilisation, adherence, take-up rates, take up rates, patients drop-out rate

### Study Selection

**Figure 1:**
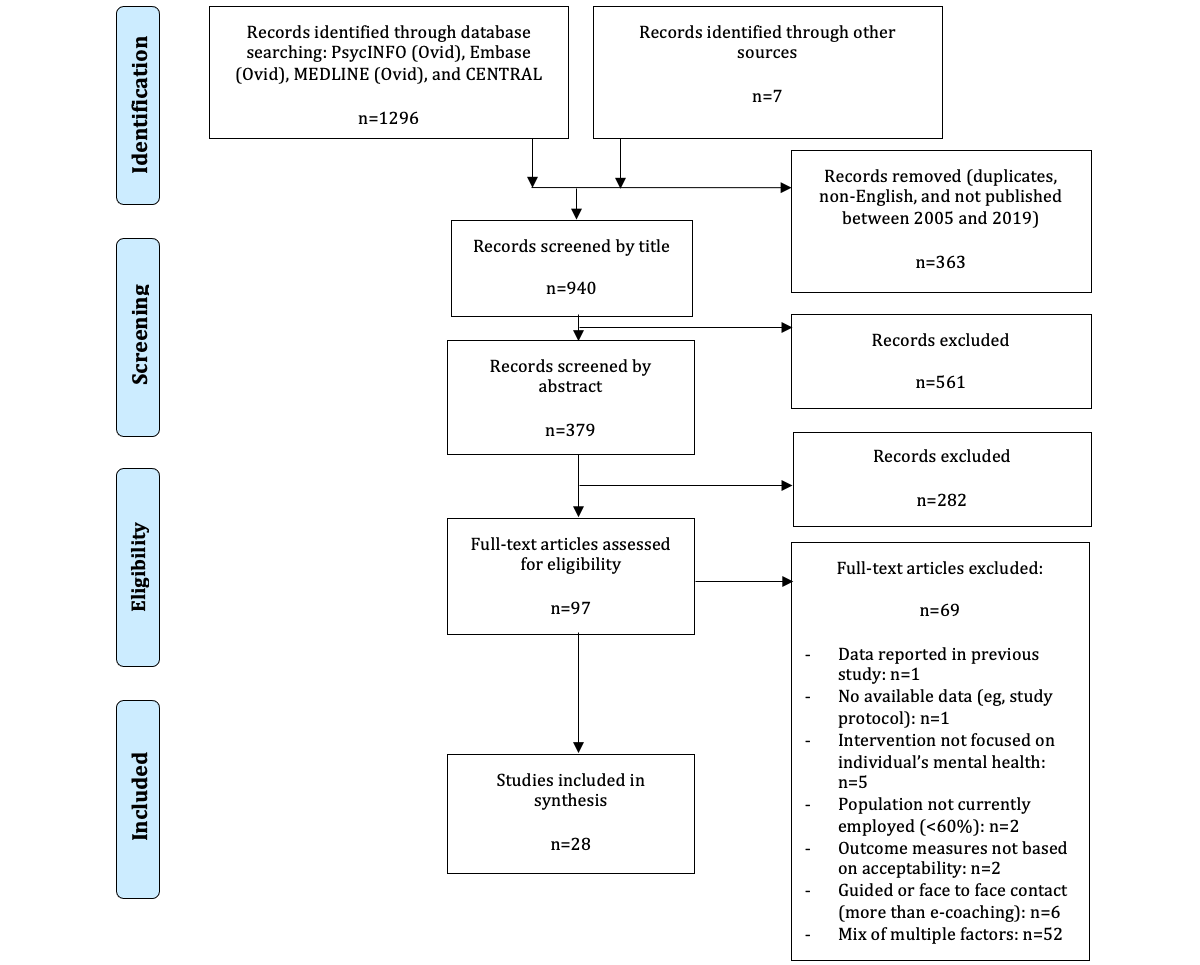
PRISMA (Preferred Reporting Items for Systematic Reviews and Meta-Analyses) flow diagram.

Duplicates were removed, and titles, abstracts, and full texts were scanned for the inclusion criteria. After the assessment of the full texts’ eligibility by the first author (JS), all the included studies were summarized and synthesized. The study selection process is outlined in the PRISMA flowchart (Figure 1).

### Data Collection Process

Data were collected according to the following criteria: reference, characteristics of the intervention, its aims and objectives, study design, population, setting and recruitment, results, acceptability, and—if available—reasons for dropout, as well as qualitative data.

### Quality Assessment

The quality of the studies was assessed using the Critical Appraisal Skills Programme (CASP) checklist [20] for both qualitative studies and RCTs. Studies were evaluated based on research design, representativeness, recruitment procedure, presence of a comparison group, dropout rate, validity, reliability, and relevance of the measurement tools. Quality was graded with a traffic light system based on the 10 quality questions of the CASP and answered with *yes* (green) if the information was present, *no information* (red), and *not available* (yellow) if the information was not apparent or clearly outlined within the study (Multimedia Appendix 1 [21-48]). Studies were included in this systematic review irrespective of quality judgment.

### Synthesis of Data

Similar to corresponding systematic reviews on the acceptability of web-based interventions [10], the level of acceptability was categorized into the following quartiles: low (− −), moderate (−), high (+), and very high (+ +). This was specifically used for studies that reported a satisfaction rating on a scale, the percentage results of which could then be transferred to the suggested levels of acceptability. In addition, studies reporting dropout rates and compliance percentages were organized according to the 4-quartile rating system for acceptability. If studies reported mixed results, including positive and negative outcomes on different acceptability factors, they were rated with a tilde (~). Qualitative studies were synthesized in an integrative, meta-aggregative style following methodological guidance on the use of meta-aggregations [49] as well as using similar systematic reviews [50]. The key data were extracted and are outlined in Table 1 (see Multimedia Appendix 2 [31-33,35,38,39,45-48] for direct measures and qualitative data sources).

**Table 1:** Study characteristics^a^.

Author and country	Intervention, duration, and aim	Design and recruitment	Population					Results		Acceptance measure		Reasons for dropout		Level (acceptance)
			Sample size, N	Age (years)	Gender	Employment details								
Abbott et al [42], Australia	Internet-delivered CBT^b^ program for employees with tinnitus distress in industrial organizations; 6 weeks; effectiveness of the program	Clustered RCT^c^ comparing CBT intervention group with IOC^d^; recruited in industrial organizations (BP Australia and BHP Billiton)	56	CBT: mean 50.5 (SD 9.5); IOC: mean 48.7 (SD 8.6)	CBT: 96% men; IOC: 82% men	—^e^	CBT program was similarly effective to the information program for treating tinnitus distress, depression, anxiety, stress, and quality of life		Attrition rate: 50% (CBT: 70%); satisfaction: 73.4% (mean 5.14/7)		Unknown		~^f^	
Allexandre et al [21], United States	Web-based interactive educational stress management program (website), *Stress Free Now*, using mindfulness meditation; 8 weeks; effectiveness of the intervention	RCT comparing 4 groups, including no support, group support, group and expert clinical support, and waitlist control; recruitment via email in a corporate call center	161	Mean 40.0 (SD 12.6)	83.2% women	49.1% full-time work shift (days); debt collectors and customer service or fraud representatives	Participants favored guided practices and showed low use of program. All groups decreased in perceived stress and improved in psychological and emotional well-being		Web-based use: 10% to 15% (intervention)		Lack of time		− −^g^	
Beiwinkel et al [36], Germany	Web-based program, *HelpID*, for depression based on CBT and awareness training; 12 weeks; effectiveness of *HelpID* in reducing sickness absence and depression	RCT comparing intervention with control; recruitment via health insurance	180	Mean 48	68% women	51% full-time work	*HelpID* effectively reduced depressive symptoms		Dropout: 45.5% after the assessment, 67.7% follow-up; satisfaction: 68.2% (mean 2.04, intervention)		Relationship between age (older) and education (higher) and dropout rates (lower)		−^h^	
Birney et al [37], United States	Mobile app intervention *MoodHacker*, CBT-based depression self-management; 6 to 10 weeks; effectiveness of a program to reduce stress and prevent depression, anxiety, and substance abuse among employees	RCT: MoodHacker group compared with alternative care with links to websites on depression; recruited via EAPs^i^ and other outreaches	300	MoodHacker: mean 40.6 (SD 11.5); alternative care: mean 40.7 (SD 11.2)	MoodHacker: 74.6% women; alternative care: 78.7% women	56% full-time, 35.3% part-time, and 8.7% self-employed	MoodHacker caused significant effects on depression symptoms compared with alternative care		Attrition: 6.7% follow-up; satisfaction: 76% (mean 4.6/6); system usability: B+		Unknown		**+ +^j^**	
Billings et al [22], United States	Web-based stress and mood management multimedia program for employees based on CBT; 12-week access; effectiveness of the program to reduce depression and increase behavioral activation, knowledge of depression, and performance at work	RCT: experimental and control; recruited from a technology company via email and health fair	309	Most (51%) between 30 and 40	70.6% women	—	Decrease in stress, increase in knowledge of anxiety and depression as well as positive perception of treatment and improvement in the consumption of alcohol; most used it only once		Ratings (0-5): 71% useful (mean 3.55); interesting: M 3.47; appealing: M 3.34; motivating: M 3.21		Participants who knew how to handle mood and stress at baseline had an increased likelihood of finishing the study		**+^k^**	
Bolier et al [23], the Netherlands	Web-based health promotion programs (*Colour Your Life, Don't Panic Online, Drinking Less, Psyfit*, and *Strong at work*) designed for the work setting aiming to decrease stress and prevent substance abuse, depression, and anxiety in health professionals; 6 to 12 weeks; measure effectiveness of the modules	Clustered RCT: web-based condition and waitlist control; recruited nurses and health professionals in a medical center via mail	1140	Mean 40	79.8% women	71.9% nurses	The intervention significantly enhanced positive mental health		Uptake rate and compliance: 16% logged in, 5% started; dropout: 60.7% (intervention), 44% overall		Age predicted dropout (the younger the participants, the more likely they were to drop out)		− −	
Ebert et al [40], Germany	Web-based unguided recovery training, *GET.ON*, for teachers with insomnia and psychological detachment from the workplace; 6 weeks; psychological efficacy of *GET.ON*	RCT: intervention and waitlist control group; recruited via email at schools by the Ministry of Education (Germany, NRW^l^)	64	Mean 48.5 (SD 9.9)	74.2% women	—	Significant reduction in insomnia severity		Completion rate: 48.4% all sessions		38.5% technical problems, lack of time or motivation, disputed usefulness, or did not see any more benefit in using the program further before the final module; others did not report reasons		−	
Ebert et al [24], Germany	Unguided web-based stress management program, *GET.ON Stress*, for employees using problem solving and emotional regulation; 7 weeks; efficacy of the program	RCT: intervention or waitlist control; recruited from general employees via the occupational health program of a health insurance company as well as via contacted HR^m^ departments in Germany	264	Mean 42	72% women	75% full-time; diverse sectors including economy, health, service, and social	Effectively reduced symptoms of mental and work-related stress among employees with stress		Attrition rate: 42% (7 sessions); dropout: 90% provided follow-up data; satisfaction (high): 95% overall		—		**+ +**	
Hamamura et al [25], Japan	Computer-delivered intervention (app), *Self-Record*, that facilitates cognitive restructuring for distress and alcohol consumption through self-monitoring of thought and activities; 4 weeks; effectiveness of the intervention on mental distress and consumption of alcohol	Pilot non-RCT, quasi-experiment with intervention and control groups; recruited via research marketing company	557	Mean 38.82 (SD 9.58)	41.2% women	71.6% employed by a company, 7.5% employed by the government or a nonprofit organization, 6.3% self-employed, and 3.1% professionals	Intervention heightened participants’ perception of their pathological thoughts and alcohol consumption, whereas they only decreased face to face		Dropout rate: 15.3% follow up; adherence: 64.8% (intervention) stopped after the first day		—		−	
Heber et al [26], Germany	Stress website, *GET.ON*, including psychoeducation and interactive exercises tailored through personalized feedback; 4 weeks; efficacy of the intervention	RCT: intervention and waitlist control group; recruited by the Ministry of Education from the general working population showing symptoms of stress and through newspaper articles	264	Mean 43.3 (SD 10.2)	73.1% women	77.3% full-time	Web-based interventions effectively decreased stress in employees		Completion rate: 70.05% all sessions; satisfaction (high): 92.2%		Time constraints (4/9), motivation constraints (3/9), technical difficulties (1/9), and dissatisfaction with the intervention (1/9)		**+/++**	
Ketelaar et al [27], the Netherlands	eMH^n^ interventions for health professionals—*Psyfit*, *Strain at work*, *Colour your life*, *Don’t panic online*, and *Drinking less*: self-help on the internet (CBT and other); evaluate eMH approach targeting work functioning and psychological well-being	RCT, randomization at ward level with intervention and control groups; recruited nurses and health professionals employed at an academic hospital	1140	Mean 39.5	80% men	—	eMH approach was not more effective than a control to increase work functioning and psychological well-being		Compliance rate: 6% started the intervention; dropout: 45% to follow-up		Younger participants were more likely to drop out; technical problems		− −	
Ly et al [28], Sweden	Mobile phone stress management intervention for managers including short audio lectures, information, and exercise focusing on acceptance and commitment therapy; 6 weeks; efficacy of the smartphone treatment	RCT: stress intervention and waitlist control group; recruitment took place after a presentation about the project at multiple organizations (Swedish or American) and via advertisements on the internet	73	Mean 41.5 (SD 7.2)	57.5% men	—	Intervention reduced stress and increased general health among managers		Adherence: 44.4%		—		−	
Nevedal et al [43], United States	Digital health coaching program for chronic pain management using psychoeducation on self-management, coping, and stress; 4 weeks; effectiveness of the program on work interference, activity, stress, pain, quality of life, and health	Case report; 1-group design; recruited via mailings, emails, and posted communications within 37 American organizations or a member of 1 of 18 health care plans	645	Mean 56.16 (SD 12.83)	69.3% women	—	Supported effectiveness of interventions on pain (1 and 6 months after treatment) as well as quality of life (after 6 months)		Satisfaction (good or better): 82.6%		—		**+ +**	
Feicht et al [44], Germany	Happiness exercises to develop a positive psychological state; 7 weeks; examined the impact of the intervention on psychological and physiological parameters	Longitudinal design (2 groups—intervention and control); recruited via local insurance company in Germany (2 participating departments were chosen by the company)	142	Mean 37 (SD 7.7)	68.8% women	—	Happiness, satisfaction, mindfulness, and quality of life improved; stress decreased; and recovery experience increased significantly		Dropout rate: 31.3% total		Difference in age in intervention and control groups (10 years)		**+**	
Thiart et al [29], Germany	Internet-based CBT-I^o^ intervention, *GET.ON Recovery*, for stress, work-related strain, and insomnia in teachers; 6 weeks; evaluate the efficacy of the intervention	RCT: intervention group and waitlist control; recruited via email sent to schools in Germany	128	Mean 48.0 (SD 9.9)	74.2% women	100% school teachers	The intervention reduced sleep difficulties and fostered psychological detachment from work		Completion rate: 95.3%; satisfaction: 91% would recommend it		—		**+ +**	
Umanodan et al [30], Japan	Computer-based stress management training using self-paced behavioral, communication, and cognitive techniques; 7 weeks; effectiveness of the program in improving mental health and performance at work	Clustered RCT; recruited via informational posters and the supervisor during meetings in a manufacturing company	263	Mean 38.85	92.6% men	23% managers	Knowledge about stress management and coping skills increased (if participants had enough time)		Completion rate (intervention group): 89%		High baseline levels of distress increased the chance of dropout		**+ +**	
Wood et al [41], United States	Resilience mobile app to decrease burnout (assessment tools); 4 weeks; assess usability, acceptability, and effectiveness	Pilot study; recruited mental health care professionals from a health care system	30	Mean 42.5 (SD 12)	—	43% psychologists, 30% social workers, 13% psychiatric nurses, 7% psychiatrists, and 7% other	App reduced burnout and compassion fatigue in participants		System usability (overall): 79.4%		—		**+ +**	
Bush et al [31], United States	*T2 Mood Tracker* mobile app to track symptoms associated with deployment-related behavioral health issues (well-being, anxiety, stress, PTSD^p^, injury, and depression); 1.4 weeks; assessment of the utility of the app	Mixed methods design: qualitative and quantitative (Likert-style and open-ended questions); recruited via posters and flyers distributed by WTU^q^	8	—	62% men	Clinical social work staff	The app was perceived as easy to use, helpful, and beneficial		Useful rating: 88%; qualitative: utility rating positive but could incorporate additional factors to make it more manifold		2 participants did not want to download the app (privacy concerns were assumed)		**+ +**	
Carolan et al [32], United Kingdom	Web-based stress management intervention, *WorkGuru* (CBT, mindfulness, and problem solving); 8 weeks; employees’ attitude toward digital mental health interventions at work	Qualitative study: 18 semistructured interviews (taken from previous RCT with and without access to a web-facilitated discussion group); recruited from 6 UK-based organizations and invited via mail (universities, local authorities, third sector, and telecommunications)	18 (based on the sample N=82)	Mean 45	78% women	78% office work and 22% mixture of office and client work	Outlined advantages of digital mental health interventions, but high barriers appeared with the application in the workplace		Engagement: 39%; qualitative: preference for short, interactive, easy to use, personalized, and anonymous interventions and access via computer or mobile phone		—		−	
Deady et al [48], Australia	Emergency service workers’ attitude toward mobile mental health apps	Cross-sectional study; recruited from 4 metropolitan Fire and Rescue stations	106	Mean 37.8 (SD 9.51)	88% men	Firefighters	Participants showed positive perception and interest in using mental health apps but had preferences regarding language, features, and therapeutic techniques		Divided interests in using a mental health app. Apps should avoid stigmatized terminology and focus on well-being, mental fitness, resilience, stress, lifestyle, and sleep by implementing attractive multimedia features		—		**+**	
Deady et al [38], Australia	Acceptance and effectiveness study on *HeadGear*, an app-based program aiming to decrease depressive symptoms and increase well-being; 5 weeks	2-stage pilot study; recruited via email and Facebook from industrial organizations (agriculture, freight or postage, and mining)	Stage 1: 21; stage 2: 84	Stage 1: mean 37.86 (SD 10.98); stage 2: mean 38 (SD 9.23)	Stage 1: 50% women; stage 2: 100% men	Stage 1: most worked in freight and postage (n=11); stage 2: male-dominated industry	HeadGear was effective and reduced symptoms significantly. However, attrition rate was high		Utility: 40% to 50% would use it; qualitative: most appreciated the utility, helpfulness, overall ease, and accessibility but complained about engagement and navigation issues		—		−	
Eklund et al [33], Sweden	University staff’s experiences of a customized, interactive, web-based program that aims to change behavior in stress management as well as explore intervention adjustments	Explorative qualitative study: semistructured interviews; recruitment via 3 departments at the university	9	Mean 45.9	—	University staff	Staff accepted a web-based program for stress-related problems		Acceptance was positive as long as it was short in time and applied in a transparent and tailored way		—		**+**	
Hennemann et al [34], Germany	Employees’ acceptance of organizational eMH interventions focusing on work-related distress	Longitudinal cohort study: self-administered questionnaire; recruited employees showing health problems and previous sickness absence	1829	Mean 49.93 (SD 4.06)	—	—	Attitudes toward organizational eMH interventions were disadvantageous		Acceptance (low): 89.1%; suggestions for improvement of acceptance: previous education (awareness and attitudes regarding efficacy and usability)		Higher scores in men and high-education group, those with previous experience with eHealth, and mentally demanding work types; lower scores in those diagnosed with a mental health disorder and non–internet users		− −	
Peters et al [45], Australia	Explorative workshop of perceptions, thoughts, and preferences of employees in male-dominated workplaces to build and adapt a mental health mobile app	Exploratory qualitative study; recruited via emails distributed to 2 organizations (state fire and rescue service and a freight transport organization)	60	Between 26 and 65	92% men	27% rural, 23% suburban, and 50% urban	Relevance of considering language use and preferred features and balancing preferences with the need for evidence-based interventions		Men preferred unstigmatized language use, a simple mood management app, and guidance involvement		—		**~**	
Schneider et al [39], United Kingdom	Views and acceptance of 2 self-help applications for depression: *MoodGYM* (cCBT^r^) and informational websites applied at work; 5 weeks	Mixed methods; recruited from 3 organizations: 2 private enterprises (telecommunications and transport) and 1 health organization	637	Mean 42 (SD 9.6)	50.2% men	—	Evidence-based computerized approaches supported acceptability, which could be increased by taking care of barriers and users’ expectations		Dropout: 63%; positive rating: 24%; various intrinsic and extrinsic barriers that lead to a high unacceptance; acceptance increases with interactive support		Intrinsic: intrapersonal problems; extrinsic: technical problems; generic: perception of cCBT		− −	
Wang and Ho [46], Canada	Explorative study on barriers and preferences for specific features among male workers in a mental health tool	Cross-sectional study; recruited by random digit-calling method to households collecting data from 511 men with risk of depression	841	Mean 44.3 (SD 13.7)	100% men	—	Overall positive results, but men’s preferences and perceived barriers should be taken into account to increase acceptability		Acceptance in men was good, but apps should be mobile and tailored to preferences, including various topics and designs		Having high risk of depression at baseline increased the chance to see the utility of the intervention compared with low-risk individuals (83.4% vs 75%)		**+**	
Williams et al [35], United States	Feasibility of a web-enhanced behavioral self-management program, *Stress Gym*, in a military setting built on the model of cognitive appraisal by Lazarus and Folkman	Cross-sectional study; recruited and invited all active-duty members at Naval Medical Center, Portsmouth, Virginia	142	Mean 41.1 (SD 9.2)	55% women	24% officers and 76% enlisted sailors	Supported the feasibility of Stress Gym as being a web-based CBT-based self-help intervention accepted by the users and demonstrated reduction in stress		StressGym was rated as very useful and informative		—		**+ +**	
Wilson et al [47], United States	Soldiers’ attitude toward technology-based approaches to mental health care	Cross-sectional study; recruited from pre- and postdeployment clinic (in the waiting room for screening visits)	352	Mean 25.9 (SD 5.8)	92% men	—	Feasibility of technology-based approaches was supported		Willingness to use: 84% were willing to use one of the 11 interventions; comfort: 75% felt neutral/very comfortable using a computer/program		—		**+ +**	

^a^Sorted from indirect to more direct measures.

^b^CBT: cognitive behavioral therapy.

^c^RCT: randomized controlled trial.

^d^IOC: information-only control.

^e^Data missing or not relevant.

^f^Mixed results.

^g^Low.

^h^Moderate.

^i^EAP: employee assistance program.

^j^Very high.

^k^High.

^l^NRW: North Rhine-Westphalia.

^m^HR: human resources.

^n^eMH: e-mental health.

^o^CBT-I: cognitive behavioral therapy for insomnia.

^p^PTSD: posttraumatic stress disorder.

^q^WTU: Warrior Transition Unit.

^r^cCBT: computerized cognitive behavioral therapy.

## Results

### Review Process

The characteristics of the studies are outlined in Table 1 as well as in Multimedia Appendix 2 including more details. Within the review process, 1303 papers were identified, of which 363 (28%) were duplicates, not published in English, or published before 2005. Of the 1303 papers, titles and abstracts were then scanned, a process that identified 940 (72%) and 379 (29%) papers, respectively. Papers were excluded if the interventions were independent of work environments, did not include most employees, and did not focus on mental health issues. Most studies were excluded owing to the involvement of face-to-face or telephone guidance by a coach or therapist. Ultimately, 28 studies were identified for further analysis, which either reported indirect measures of the acceptability of web-based interventions (n=17, 61%) or provided qualitative data on acceptability (n=11, 39%; Figure 1).

### Study Characteristics

The 28 included studies had an overall sample of 9739 participants, with sample sizes ranging from 8 to 1140. The mean age of the participants was 40.7 years, and most participants were White and employed full-time.

The studies had various differing methodological designs and study characteristics, which are outlined in Table 1 as well as in Multimedia Appendix 2 including more details on direct measures. Interventions were heterogeneous in type, application outcome focus, length, setting (within a specific organization or random employees), and characteristics of the participants (type of profession and demographics). In addition, they differed in the level of support potentially provided in web-based format (email or message) versus unguided. Most interventions (18/28, 64%) were focused on reducing stress [21-35] and depressive symptoms [23,31,36-39] in employees. Other interventions focused on insomnia (2/28, 7%) [29,40], anxiety (2/28, 7%) [23,31], panic (2/28, 7%) [23,27], psychological detachment from work (1/28, 4%) [40], resilience (burnout; 1/28, 4%) [41], mood (1/28, 4%) [22], tinnitus distress (1/28, 4%) [42], chronic pain (1/28, 4%) [43], substance misuse (2/28, 7%) [23,25], and well-being or happiness (3/28, 11%) [31,38,44]. Studies and interventions often included more than one focus of mental illness, used various treatment techniques, and assessed the acceptability of general web-based mental health interventions [34,45,46]. Cognitive behavioral therapy (CBT) was the most used form of intervention (9/28, 32%). Other interventions applied mindfulness (3/28, 11%), psychological education (3/28, 11%), cognitive appraisal or restructuring (2/28, 7%), emotional regulation (1/28, 4%), acceptance and commitment therapy (1/28, 4%), problem solving (2/28, 7%), exercise (1/28, 4%), or communicational strategies (1/28, 4%). Approximately 7% (2/28) of the studies used tracking or assessment tools for burnout and mood (eg, depression, stress, or well-being). The studies were predominantly RCTs (13/28, 46%). CBT was mostly used in interventions that tackled depression or stress at work. For example, the CBT interventions focused on depression were *HelpID*, *Mood Hacker*, *Colour Your Life*, and *MoodGym*. The interventions that used CBT and aimed to reduce stress and mental strain were *Psyfit*, *Strong at work*, *GetON Recovery*, and *Work Guru*.

Of the 28 studies, 13 (46%) were RCTs, 4 (14%) were cross-sectional studies, 3 (11%) were qualitative studies, 3 (11%) were pilot studies, 2 (7%) were longitudinal studies, 2 (7%) were mixed methods studies, and 1 (4%) was a case report. The studies mostly used waitlist control groups, internet-based information website groups, or variations of intervention type groups as comparators. The studies originated in the United States (8/28, 29%), Germany (7/28, 25%), Australia (4/28, 14%), the Netherlands (2/28, 7%), Japan (2/28, 7%), Sweden (2/28, 7%), the United Kingdom (2/28, 7%), and Canada (1/28, 4%).

### Methodological Quality

The methodological quality of the studies is summarized in Multimedia Appendix 1. The studies were assessed for quality using the CASP [20] qualitative and quantitative templates and reported in the form of a traffic light schema. Various quality flaws were outlined in the studies, and no study met all 10 criteria marked by the CASP [20]. Independent of quality, all studies (28/28, 100%) were included in the final synthesis. Allocation bias appeared to be low in the quantitative studies as participants were mostly randomly distributed to their condition (15/17, 88%). However, qualitative studies often indicated performance and detection bias as studies often missed reporting on blinding status or researchers’ awareness of the participants’ condition. Selection bias was generally high as participants repeatedly originated from specific population samples (eg, male-dominated industry workers or female educational staff). Attrition bias was predominantly high as various studies reported a high dropout rate, which weakened their generalizability. Several studies missed reporting on the specific demographics of their samples and, thus, might risk the presence of confounders, whereas other studies (4/28, 14%) clearly outlined their risk of confounding [25,26,30,42]. The analysis of quantitative studies was generally good as all studies used data from all participant groups in their final analysis. Qualitative studies showed generally good quality in the guidance of clear questions, taking care of ethical considerations, and the provision of clear information on methodology. However, various studies missed accounting for the potential bias caused by the relationship between the researchers and the participants.

### Setting and Types of Employees

The recruitment setting and included characteristics of the employees were very diverse (Table 1). Some studies (10/28, 36%) used the whole working population, recruiting samples via local insurance companies, occupational programs or employee assistance programs, random digit calling, or advertisements [24,26,28,34,36-38,43,44,46]. Alternatively, some studies used specific population samples originating from 1 type of profession. In particular, samples were recruited from the military (2/28, 7%) [35,47], telecommunications (2/28, 7%) [21,39], transport (2/28, 7%) [39,45], the public sector (1/28, 4%) [39], state fire and rescue services (2/28, 7%) [33,45], office and client employees (1/28, 4%) [32], university staff and teachers (3/28, 11%) [29,33,40], clinical staff and health professionals (4/28, 14%) [23,27,31,41], manufacturing and industrial workers (2/28, 7%) [30,42], marketing (1/28, 4%) [25], and technology (1/28, 4%) [22]. Most studies were conducted in the United States (8/28, 29%) and Germany (7/28, 25%). Germany primarily recruited from the general working population [26,34,36,44], whereas the United States mainly recruited from multiple specific locations, including larger organizations (eg, corporate call centers and technology companies), health centers, and military-related workplaces. Their acceptability results were mixed in outcome, ranging from very high in the study by Beiwinkel et al [36] to very low in the study by Hennemann et al [34]. The synthesis did not outline any pattern of setting and participant characteristics that was associated with the acceptability level of web-based interventions.

### Intervention Characteristics and Country of Conduct

As outlined in the *Study Characteristics* section, most studies used CBT in their administered interventions (9/28, 32%). CBT was relatively equally distributed across Western countries, including the United States (2/9, 22%), Germany (2/9, 22%), the Netherlands (2/9, 22%), the United Kingdom (2/9, 22%), and Australia (1/9, 11%). Summarizing the CBT studies, the acceptability level indicated that 44% (4/9) of the studies had a low to moderate level of acceptability, whereas 33% (3/9) of the studies showed a high to very high acceptability level. Approximately 11% (1/9) of the studies had a mix of moderate and high acceptability levels. Other analyzed intervention types (mindfulness, psychological education, cognitive appraisal, emotional regulation, acceptance and commitment therapy, problem solving, cognitive strategies, exercise, and tracking tools) did not indicate any pattern of acceptability level. Broadly speaking, the intervention type, country of conduct, and outcome of the study did not indicate any notable patterns. However, most of the studies (26/28, 93%) were conducted in Western countries.

### Measure of Acceptability

Relevant studies measured acceptability in different ways. They used direct measures of acceptability, which included qualitative data through questionnaires and interviews, or indirect quantitative measures by means of take-up, dropout, compliance, adherence, attrition, or completion rate. Some studies used both direct and indirect measures. All measures of acceptability are outlined in either Table 1 or Multimedia Appendix 2 (qualitative synthesized data) in the context of the reference, intervention, sample, study design, recruitment, outcome, indirect and direct acceptability measures, available reasons for dropout, example quotations from interviews, and an individually rated acceptability level.

### Direct Measure of Acceptability

Table 1 and Multimedia Appendix 2 present the direct outcome of employees’ acceptability of web-based therapy in the workplace. When categorizing the qualitative outcome into key themes, the following topics commonly emerged: (1) general interest in or willingness to use web-based interventions, (2) employees’ satisfaction rating of the utility of the interventions, and (3) preferred features of the design and application style of the interventions. Most participants reported a generally positive interest in and acceptability of web-based interventions [31,33,35,38,46]. However, there were mixed results and negative opinions in other studies [32,33,39,48]. Common preferred features of web-based mental health interventions were the use of nonstigmatized language [45,48], the preference for interventions with interactive support [39,45], and broad application spectrum as well as short mobile and interactive multimedia interventions [31,35,38,48]. The synthesized outputs of the studies were written in descriptions of each theme as well as provided within the context of the setting and intervention type. To deliver a deeper insight into common themes, Multimedia Appendix 2 provides quotations of interviewees in primary studies. As this systematic review synthesized key themes in an integrative, meta-aggregative way, quotations aid in the understanding of the summarized key themes. Most of the studies reported details on the satisfaction rating on a scale associated with web-based interventions. Employees were mostly satisfied with the interventions and rated their utility positively.

In addition, multiple studies assessed acceptability using satisfaction, usability, or interest ratings of the intervention (Table 1). Satisfaction ratings were frequently used in the studies [22,24,26,29,31,36-38,41,43]. The average satisfaction score was 82.6%, which is similar to a very high individual-defined acceptability level (++) of web-based interventions. Moreover, 14% (4/28) of the studies reported a score of 0.85% for practical use [22,31,38,41], equivalent to a high (+) acceptability. In particular, Wilson et al [47] reported a rate of 75% in “comfortability” of using a mental health program on the computer and an 84% rate in “willingness to use.” In contrast, Hennemann et al [34] reported that 89.1% of participants rated low on the “acceptability” of general occupational web-based mental health interventions. Both studies were very heterogeneous in intervention specificity and sample population. Hennemann et al [34] explained the negative outcome by the direct predictor variables of acceptability with “social influence,” “effort” and “performance expectancy,” and “time spent in the web” as well as the “frequency of searching online for health information.”

### Indirect Measure of Acceptability

This systematic review included hypothetical measures of acceptability characterized by dropout, attrition, compliance, adherence, uptake, and completion rate. The indirect or hypothetical measures of the acceptability of web-based interventions in the workplace are summarized in Table 1. The mean percentage of dropout rates from the included studies was 50.9% with a range of 15.3% [25] to 67.7% [36], which is equivalent to a moderate individual-defined level of acceptability [23-25,27,36,39,44]. A few studies reported the reasons for dropout or termination of the interventions. Repeated reasons were lack of time [21,26,40], technical difficulties [26,27,39,40], younger age [23,27,36], lower education [36], lack of motivation [26,40], no need for help [40], ability to manage stress personally [22], dissatisfaction with the intervention [26,39], higher initial level of psychological distress [30], and privacy concerns [31]. Other measures of acceptability included an average attrition rate of 32% [24,37,42], an average adherence rate of 54% [25,28], an uptake and intervention start rate of 11% [23,27], and a completion rate of 68% [30,40]. As visible in the outcome, there was no clear consensus in acceptability level, and the comparison of studies was difficult as they were heterogeneous in study design, sample, and methodology. However, the most frequently reported indirect measure of acceptability was the dropout rate, supported by a moderate (−) level of acceptability.

## Discussion

### Principal Findings

This systematic review assessed the levels of employees’ acceptability of web-based interventions aimed at improving mental health. The findings showed a generally positive level of acceptability and highlighted various factors to be considered in making interventions acceptable, engaging, and useful for employees. Themes to be addressed with caution when introducing interventions are the use of stigmatized terminology, including words of ill health and mental illness. In terms of implementation, applications are recommended to be short and use interactive multimedia tools.

Results were obtained from 28 separate studies. Satisfaction ratings and feedback appeared positive, particularly when the interventions included multimedia and nonstigmatizing language. In particular, 79% (22/28) of the studies showed acceptability measures from high to very high, and 54% (15/28) of the studies reported acceptability levels from low to moderate (overlap when studies reported both quantitative and qualitative results). The average satisfaction rating was >80%, and the employees rated the interventions’ utility as good overall. However, quantitative measures contradicted the universal positive perspective of web-based interventions by means of the common measured dropout rate of approximately 50%. Hence, the attrition rate was very high in multiple studies, which questions the efficacy of unguided self-applied interventions.

Collectively, these results are in line with other acceptability studies that supported the general acceptability of web-based interventions in clinical settings [51]. Various studies have outlined barriers to assessing acceptability; for example, negative results from indirect measures. In addition, complications in synthesis owing to the heterogeneity of the interventions have been repeatedly reported.

Stigma and attitudes toward mental health at work were an emerging theme. Acceptability levels may relate to the web-based interventions themselves or to the fact that the intervention relates to mental health. This is supported by other studies showing that there is fear of stigmatization when seeking support [52]. It may also be difficult to successfully implement web-based interventions within an organization as employees prefer to separate health matters and their workplace [32]. Hence, the issue around mental health and stigma, especially at work, may be strongly influenced by the organizational culture that influences the use of mental health interventions [45].

The relationship between dropout and acceptability requires further assessment to interpret the current evidence. Although dropout for web-based workplace interventions was high (the mean percentage score of the included studies was 50.9%), explorations of the reasons for this were limited. Indeed, studies have outlined that dropout rates might not be the result of disinterest in occupational web-based interventions for mental health issues but appear to be generally high in computerized interventions [53], suggesting that these interventions are not as engaging as guided or face-to-face sessions and people might not feel committed enough to complete the treatment or program. Consequently, web-based interventions should be tailored and made as interactive and attractive as possible by using animation tools, pictures, and videos, as well as made as short and simple as possible to increase engagement and decrease the likelihood of technical issues [12]. Furthermore, the findings of this study suggest that, before applying interventions in organizations, people’s needs, the environment, and the culture should be assessed; the interventions should be tailored accordingly; and awareness of the benefits and understanding of the use should be addressed.

### Strengths and Limitations

The generalizability of the findings across workplaces may be limited because of the diversity of individual workplaces; for example, their organizational culture and stigma or attitudes toward mental health. In addition, assessing for confounding variables, including recruitment, setting, intervention characteristics, and country of conduct, did not reveal significant information. However, most of the included studies were conducted in Western countries and used CBT-based interventions, which may further limit generalizability.

Assessing acceptability using indirect measures may be flawed as there could be multiple reasons for employees to stop the intervention. Specifically, dropping out of interventions could be the result of feeling rehabilitated and seeing no further benefit of using the intervention. Nevertheless, dropouts provide great insight into the acceptability of interventions, but more in-depth analyses of the reasons for dropping out should be conducted.

Analysis of the specific assessment of acceptability of occupational web-based interventions was limited because of the heterogeneity of the study designs, intervention types, sample characteristics, and conditions under which the interventions were provided to employees. The studies used data assessment techniques, including cross-sectional self-report methods, whereas the qualitative studies used small samples. Data collection and analysis biases may be observed based on the role of the researchers [54]. As qualitative acceptability results were generally higher compared with indirect measures, this further raises the question of the role of researcher bias. In addition, limitations regarding the consistent and objective measurement of acceptability in the wider literature prevent robust conclusions from being drawn. However, the inclusion and critical appraisal of qualitative studies may have added depth to the factors within the acceptability capture in this study [55].

Despite these limitations, this study offers a comprehensive insight into multiple forms of acceptability measures [56]. Using both qualitative and quantitative as well as direct and indirect measures of acceptability provided a deeper insight into the options for assessing the acceptability of interventions in general. Although this study focused on the workplace, it examined the acceptability of web-based interventions that could be applied more generally to support people’s mental health. For example, the findings could support the implementation of interventions outside of the workplace (eg, as part of clinical mental health treatments). These results might help clinicians, developers, researchers, and the health technology industry create effective and engaging tools in the future.

### Implications

In relation to workplace practice, before applying interventions, it would be beneficial to increase people’s knowledge of web-based interventions as well as assess their needs in general to improve their attitude toward interventions [13,34]. This is supported by Murray et al [57], whose study found that participants who rejected computerized treatments had significantly lower expectations of the usefulness of self-help and had general concerns, anxiety, and misunderstandings about computerized treatments. Hence, acceptability may be increased by identifying and correcting misperceptions before participation. Similarly, tailoring interventions to the environment and employees’ needs could increase their general interest and willingness to use them [13]. In other words, web-based interventions for employees should be adapted to the specific environment applied as well as to the users’ needs to increase engagement and acceptability levels. Generally, the acceptability of interventions might increase if employees and organizations are made aware of evidence-based web-based interventions that have multiple practical benefits and the potential to increase individuals’ mental health and well-being in the long run. Finally, the ability of web-based interventions to engage and retain users is critical for ensuring reduced dropout and increased acceptability.

Regarding future research, the results of acceptability studies could be influenced by the general stigma on mental health topics and interventions. Therefore, future research should incorporate acceptability measures of mental health issues into their analysis to assess for confounding variables. Second, regarding quantitative data on acceptability, it would be beneficial if future research included a more in-depth analysis of the reasons for dropout or attrition rates. Third, future research should also address the conceptual and methodological limitations of the research in the field. If there were more organizations using mental health interventions from various settings, the research analysis could be more homogeneous. Organizations might lack the knowledge on how to apply personal health support but could provide their employees with interventions that range from broader aspects of stress management to specified apps that tackle specific mental health issues (eg, depression). Finally, this research was conducted before the COVID-19 pandemic, which changed work styles and environments and affected how people sought and received mental health support. Further research should analyze changes in acceptability as a result of the pandemic to examine shifts in use and acceptability of mental health interventions both within and outside of the workplace.

### Conclusions

This study assessed the area of acceptability of web-based workplace interventions for mental health. In general, workers are open to web-based mental health interventions. However, qualitative and quantitative studies suggested varying levels of acceptability, raising the possibility of bias. The importance of stigma, organizational culture, and the implementation of the intervention were highlighted, the latter relating to the engaging design and quality of the intervention as well as the approach to delivery in the workplace itself. Several factors were identified that need to be considered to ensure the effective implementation of web-based interventions in the workplace, some aspects of which may also apply to the general use in supporting people’s mental health. Interventions should be tailored to the respective individual needs and cultural context, use nonstigmatized language, and be made interactive and easy to use. It is also recommended to foster an understanding of the potential value of an intervention to increase its acceptability. Methodological limitations were highlighted to guide the cautious interpretation and generalization of early evidence in this area along with the need to improve methodological rigor in emerging research.

## Appendix

Multimedia Appendix 1 Quality rating.

Multimedia Appendix 2 Direct measures.

## Abbreviations

CASP: Critical Appraisal Skills Programme
CBT: cognitive behavioral therapy
ENTREQ: Enhancing the Transparency in Reporting the Synthesis of Qualitative Research
PRISMA: Preferred Reporting Items for Systematic Reviews and Meta-Analyses
RCT: randomized controlled trial
