# Supplementary figures and images for: Acceptability of Web-Based Mental Health Interventions in the Workplace: Systematic Review
Source: JMIR Ment Health. 2022 May 11;9(5):e34655. doi: 10.2196/34655 (PMC9133994; doi:10.2196/34655)

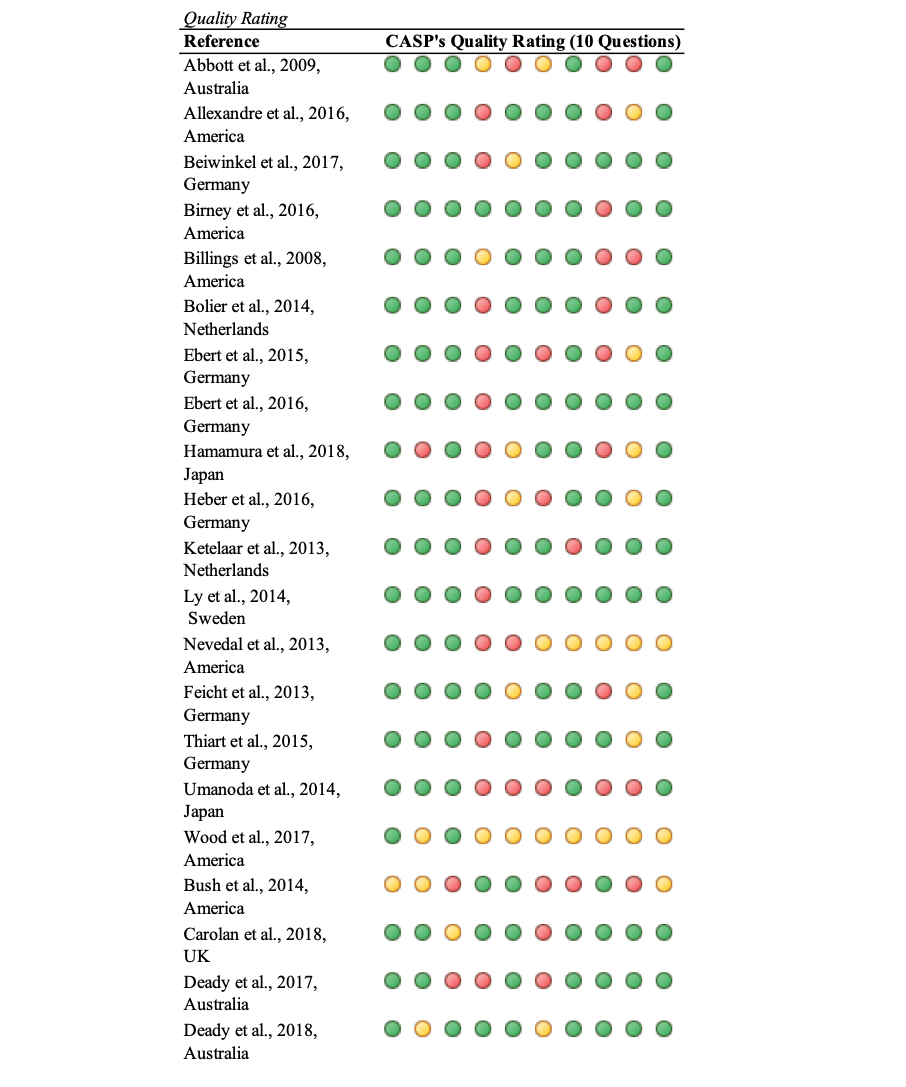

Supplement: Multimedia Appendix 1 [file mental_v9i5e34655_app1.png]
