# Supplementary material for: Acceptability of Web-Based Mental Health Interventions in the Workplace: Systematic Review
Source: JMIR Ment Health. 2022 May 11;9(5):e34655. doi: 10.2196/34655 (PMC9133994; doi:10.2196/34655)
Supplement: Multimedia Appendix 2 [file mental_v9i5e34655_app2.docx]

### Multimedia Appendix 2

*Direct measures*

| Reference | Acceptance Measures | Category of participants view | Synthesized findings | Available key quotations of participants |
| --- | --- | --- | --- | --- |
| Bush et al., 2014, America | ﻿ *Positive* *ratings:* - ﻿Generally positive about the utility of T2 Mood Tracker - Rated as useful by most users and commented that the app would be particularly useful within the behavioral health system.  - One user found the multimedia capability of the app especially useful.  - A number of participants created and used new rating scales to fit their additional individual issues.  *Other measures:*  - 88% perceived the tracker as useful and beneficial  - 63% would use the app in the future  - 75% would recommend it to others  - 75% would use the app according to its aims | - Positive utility rating and useful within the setting and appreciated the multimedia capability of the app  - Add factors which they appreciated (migraine, nutrition, weight change, mediation, social work counselling) | Utility of program is good but could include additional factors to make it more manifold | *"Much better than social surveys that make you recall mood for last month”  “Graphs were superb.” “during a SM’s stay at the WTU”;*  *“for clients or patients seeing BH providers, right before appt time”;*  *“for social work and behavior management”;*  *“for behavior health”;*  *“for psychiatric and all of behavioral health providers”;*  *“for tracking mood to share with provider” “Used voice function for documenting because of my tremor*” (p. 1455) |
| Carolan et al., 2018, UK | *Positive:*  - Described it as convenient (free choice of accessing time and place).  - Simple, interactive, and easy-to-navigate website  - High stigma was reported to mental health - Rated the anonymity as beneficial to overcome stigma  - Benefits in access of treatment without appointment and the ability to use it in the workplace  *Negative:*  - Undefined times to use the intervention  - No private place for the treatment  - Mixing therapeutic environment with stressful work setting  - Lack of human interaction  *Engaging rating:*  - Program content and design  - Interactive  - Reminders to use it  *Barriers to use it:*  - Lack of time as key factor,  - Mental ill health as barrier  - Divided opinion on support of e-coach  *Preferences*  - Simple, interactive, easy to navigate, access from computer as well as advantage with mobile phone due to anonymity, confidentiality, and personalization  *Other measures*:  - 39% engaged with the intervention | *Appreciated factors:*  - Convenience due to anonymity and flexibility of access  - Engagement was rated as positive through content and interactivity  *Negative aspects:*  -Utility based on unspecified time of usage, place to use, mix of work with therapy, and missing therapeutic support  - Barriers through lack of time, mental ill health (lack of motivation) - Conflicting result on guidance  *Preferences for utility*:  -Simple, interactive, easy to navigate, anonymity, confidentiality, and personalization, mobile application | Various positive factors associated with computer based and mobile phone interventions including anonymity, privacy, flexible availability, and interactive. But also outlined negative factors which including the limited time and boundary between work and therapy. To make interventions acceptable they should be short, interactive mobile/computer application which are easy to use and tailored to the individual. | *﻿"Whenever I need something, I can just straight away go there without waiting for someone, waiting for an appointment or like. I can get help as soon as possible and I can get it anywhere because it’s online on the Internet". "﻿I wouldn’t tell it to anyone in my workplace". "﻿Personally it was easier to say, “I’m doing something to help myself,” but without actually having to speak to someone. You know it’s quite daunting if you’ve got a worry to actually pick up the phone and speak to someone". "﻿It’s good not to have to do things in a certain time but it’s also not good because you can often think “Actually I’ll do it later,” and never get round to it. [...] If it’s online it’s down to the individual themselves to go and do what they are required to do". ﻿"And the other problem is sitting in an open plan, hot-desking space. In our room each desk runs into the next desk, there are no privacy screens between them. So I don’t know if there’s a sense of feeling that other colleagues can see what you’re working on, they can see the screen of your computer".  "﻿Probably at the time, um I was very low, very depressed. [...] I suppose time would’ve been a bit of an issue, coupled with depression. I didn’t have any motivation at all"* (pp. 9-21) |
| Deady et al., 2017, Australia | Survey: - 64% were likely/very likely to try app - 54% interested in trying app - 74 % use it if they feel down  - 6% would never use it - 42% preferred face-to face support - 17% preferred app  - 95% confidentiality/privacy and stress/wellbeing were most important feature.  - 94% Sleep/exercise/lifestyle  - 89%resilience  - 75% rated that it is important that the app be industry specific  - 77% rate that should be separate from employer  - 76% wanted mix of audio/visual/text content would prefer wellbeing and mental fitness apps for daily wellbeing apps;  - Avoid stigmatized language | - Preferred features and advantages including mental fitness/wellbeing, wellbeing, resilience, stress, emotional wellbeing. Also, they wanted it to be tailored to the profession and separated from their employer.  - Specific features were stress/wellbeing, sleep, exercise, lifestyle and resilience applied with a mix of audio, visual and text, as well as a behavioral therapeutic approach  - To avoid stigma around mental health preferred terms like fitness, wellbeing | There is a divided interest in using mental health apps. Applications should prevent stigmatized terminology and implement focuses on wellbeing, mental fitness, resilience, stress, lifestyle, and sleep by using attractive multimedia features. | n/a |
| Deady et al, 2018, Australia | *Satisfaction:*  - 92% found it easy to use & easy to understand - 67% satisfied  - 75% recommend it to a friend ﻿ - 90% respondents it helped improve their mental fitness, and user feedback was positive.  *Attrition:*  - 40,5% was high across the stages:  *Negative:*  - Only 1 user reported substantial negative responses.  *Utility:*  - 40 to 50% claiming they would use it (often)  - 42% claiming the app met their expectation (with few disagreements)  STAGE 2: ﻿  - 40.5% completed follow-up questionnaire; ﻿  - 76% of the respondents found the app to be mostly/completely appropriate  - 90% claimed it helped them improve their mental  - 90% found it mostly/completely understandable.  Best and worst features of the app (stability, speed, look and feel, functionality, navigation, content, and other):  - 46% Content was the most popular feature reported  - 23% look, feel, and functionality Issue:  - 23% Navigation  - Engagement and commitment | - Most of the participants found the intervention good and met their expectations.  - Only half of them would use the app and many stopped using the intervention.  - Preferred features were the content and negatively seen was the navigation as well as engagement and commitment | Generally, the majority of the participants appreciated the utility, helpfulness, and overall ease and acceptability of use of the HeadGear app but complained about engagement and navigation issues. | *"﻿Improved my focus to make mindfulness a more consistent part of my day".  "Great app has really helped me look at all aspects of my life: work, relationships, interests, exercise, diet and mindfulness. This app has helped me manage my anxiety and depression". "﻿I wasn't able to sustain engagement with it. This was mostly through having some really good days. My mental health is constantly fluctuating. I think the content I saw was really good and I think if I had the time (didn't work so much) and was in a worse way [sic] would've used it more consistently".  "﻿Disengaged from longer sessions, feeling like I wasn't acting on set actions without consequence" "﻿I didn’t make enough time to complete it, (You need to) Break up long sessions/ (have) time limited options"* (pp.6-9) |
| Erklund et al., 2019, Sweden | The program was perceived as supporting stress management but also perceived as extensive and time consuming.  The theme was divided into two categories: *Defining the needs:* where the users expressed what they needed from the program and their everyday environment, to be able to use the program (﻿Time – a paradox in stress training), adequate presentation for a stressed individual, relevant program,  *-It is about me:* where the program was ﻿described as helping the users understand their own stress (﻿Taking departure from me, Understands what I need, Perceived gains, Possibilities of choices and structure) | *2 Key concepts*  - Time restriction as negative, therefore defined needs for the program to make it applicable and useful for stress management - Require the program to help them understand their own stress and transparency of treatment and outcome | The experience and acceptance of a tailored web-based application for stress-management outlined to be positive as long as it was short in time and applied in a transparent and customized way. | *"﻿It is a little contradictory to have yet one more task [the stress management program] to do when you are stressed. It is the paradox of it all". "Well, you got something [the ABC-model] to take departure from (…). What is my point A and where am I going with this"* (pp. 43-45) |
| Peters et al., 2018, Australia | ﻿*Valued tools and characteristics:*  - Mood tracker, self-assessment, and mood-fix tool  - Brevity of interactions,  - Minimal on-screen text  - Solutions-oriented approach ﻿  - Mood tracking  - Mood-fix (for quick fixes of negative mental states)  - Easy access to urgent help  - Links to mental health support organizations (external and employer-provided)  - Guidance on how to deal with specific situations that occur on the job  - Stories of lived experience from respected members of the community  - Notifications and reminders to use the app  - A self-assessment tool  - Solutions or strategies based on the self-assessment  - Progress visibility  - Guidance on how to have mental health discussions  - Brain games or puzzles  - A dashboard (showing progress and stats)  -Notifications alerting to fresh content  *Negative terminology*:  - Stress/feeling stressed,  - Struggling  - Not yourself  - Feeling shithouse  - having a shit day  - Depressed  - Feeling a bit “How ya’ going”  - Having negative thoughts and behaviors  - “mental health” was highly stigmatized and disliked by participants.  *Positive terms*:  - Change your mindset  - Relax and release  - Just get it all out  - Mood boost  - Motivators | Feature preferences for a mental health app focused on:  - Mood management  - Positive language use  - Connection to support and guidance - Simple and solution-oriented application | Men preferred non-stigmatized language use, simple mood management application, and the involvement of guidance. | *"﻿So that people can have visual feedback at the end of every week, month, cause most people won’t track back, they’ll just go, “I feel like shit,” but they won’t necessarily track back and go “this is why I feel like shit” …When you get visual things you might go “Oh, well actually, maybe I’ve had a lot of stuff going on--maybe it’s not that weird that I’m not feeling great now.” "﻿Mood boost…trying to get yourself back into a happy place" "﻿I like graphic sort-of-things..." ﻿"I think the more serious you make the issue, the more stigma there is attached to it. Like if you break down and you can have guys talking about it on a colloquial level, then more people are going to be likely to engage with it. If you make it out to be a very like “oh, he has mental health problems, that’s a big deal” people are going to step away from it in a big way "﻿Hard hitting questions to identify whether your mind is where you think it is" ﻿"What if, as part of the app, you had coaching techniques on..." "﻿[The app’s] not for somebody who's in the process of severe mental problems. This is more along the lines of getting it before it happens".* (pp. 9-15) |
| Schneider et al., 2014, UK | *Barriers:*  - Intrinsic barriers: use computer as stress reduction seen as negative as it is a work tool  - Online is familiar and convenient  - lack of time and distraction;  *Extrinsic barriers:*  - Practical obstacles to access (technical issues, issues due to dyslexia;  *Generic Judgement:*  - Critical comment 23% of all comments concerned the impersonal nature of the online approach  *Other measures:*  - 63% drop out at week 12  - 12,5% actively preferred an indirect approach  - 15,4% rated the anonymity as an advantage  - 8% easy access  *Overall:*  - 24% rated it positive, while 23% found it unacceptably/impersonal  - 60% rated online therapy to be at least as acceptable as seeing a professional (more if they used the interactive program (MoodGYM) than informational website alone | ﻿*Barriers:*  - Intrinsic: intrapersonal problems (unfortunate to reduce stress with computer, time lack) - Extrinsic technical problems;  - Generic issues: mostly pertaining to perceptions of cCBT;  specific issues about the intervention or control condition (impersonal in nature)  *Positive effects*: Liked the easiness to use it | Using computerized treatment for depression is has various intrinsic and extrinsic barriers which lead to a high unacceptance. Acceptance increases with interactive support. | *﻿“I like the fact that I have a written record and can look back on the information. The tips and relaxing techniques give me a little bit of control in working towards a feeling of well-being.” ﻿“I did not find this tool helpful. The wording (perhaps translation) was poor, sounded pat…”  ﻿“It is a fantastic idea and the only thing that has really helped me” ﻿“I have found this programme completely useless”* (p. 9) |
| Wang et al., 2017, Canada | ﻿- 69% Likely use of e-mental health programs  *Preferred features assessed:* - 61.3% information about improving sleep hygiene - 59.5% practical and exercise to help reduce symptoms of stress and depression  - 57.8% access to quality information and resources about work stress issues  *Preferred designs:* - Information about improving sleep hygiene (61,3% high risk) /(54.9 % low risk) - Practice and exercise to reduce stress (59,5%)/(38,8%)  - Quality information about work stress (57.8)/(44,2%) - Setting personal goals and track them (54.6)/(38,8%) - Watching videos online on how to deal with work and stress issues (53.3%)/ (37,9%) - Being able to access a program via mobile phone or as an app (52%)/(38,6%) - Direct referral to health professional to deal with work and stress issues in person (51.7%)/(33%) - Self-help interactive program that provides info about work problems (47.8%)/(29,4%) - Being able to chart and track your mood (42.6%)/(28.5%) - A risk calculator predicting future risk of having major depression (42.6%) /(28.8%) - Access by phone to a trained coach to help with work stress (41.3%)/ (29.6%) - Information about anger management (41.5%)/(26.8%) - Receiving printed materials (32.7%)/(23.7) - Information delivered in game format (30.7%)/ (18.8%) - Online peer connection (27.5) /(18.5%)  - Online chat room (25.3)/ (17.6) | - Topics which were preferred included sleep hygiene, practical exercise to reduce stress and depression and access to information  - Preferred design: information, practice and exercise, qualitative information, videos as examples, access via phone, direct referral to health profession | Acceptability of intervention was good among men and applications should be mobile and tailored to the preferences including various topics and designs. | n/a |
| Williams et al., 2010, America | ﻿*Positive themes:* - Positive statements about quantity of stress related info - Positive statements regarding availability of information on sleep - Positive statements about the availability of information on how to change thinking patterns, reacting to stress, relationships management | *Content:*  - Positive were the quantity of information on stress and sleep as well as on how to change thinking-pattern *Application form:*  - positive including interface, feasibility | StressGym was rated as very useful and informative | n/a |
| Wilson et al., 2008, America | ﻿*Comfort:* - 75% felt neutral to very comfortable using computer or computer program in at least  - 66% reported feeling neutral to very comfortable using a handheld device to assist mental health treatment  *Willingness to utilize* - most were neutral/willing to utilize traditional therapy and technology interventions - 33% of Soldiers who were not willing to talk to a counsellor in person were willing to utilize at least one of the technologies for mental health care - 84 % of soldiers reported they were willing to use at least 1 out of 11 technology based approaches to receive or argument care for a mental health condition | *Comfort:*  - Participants were mostly comfortable with a computer-based intervention  *Willingness to utilize*:  - Most were willing to use a computerized intervention  - Especially willing to use in cases of avoidance of face-to-face session | Generally, participants were interested and willing to use computerized interventions. | n/a |
